# Enhancing primary care and preventive services through Interprofessional practice and education

**DOI:** 10.1186/s13584-020-00371-8

**Published:** 2020-03-23

**Authors:** Terri Fowler, David Garr, Natalie Di Pietro Mager, Joan Stanley

**Affiliations:** 1 College of Nursing, Medical University of South Carolina, 99 Jonathan Lucas Street, MSC 160, Charleston, SC 29425 USA; 2 College of Medicine, Department of Family Medicine, Medical University of South Carolina, 5 Charleston Center, Suite 263, Charleston, SC 29425 USA; 3 College of Pharmacy, Ohio Northern University, 525 S Main Street, Ada, OH 45810 USA; 4 American Association of Colleges of Nursing, 655 K Street, NW, Suite 750, Washington, DC 20001 USA

**Keywords:** Preventive services, Primary care, Interprofessional practice and education

## Abstract

Interprofessional (IP) practice and education are important when seeking to respond to the growing demand for primary and preventive care services. Multiple professions with synergistic expertise are needed to effectively provide health promotion, disease prevention, and patient education and to help patients with multiple comorbidities, chronic health conditions, and care coordination. A recent study by Schor et al. titled, “Multidisciplinary work promotes preventive medicine and health education in primary care: a cross-sectional survey,” compares the implementation of preventive services in three primary care models. Higher rates of health services, patient education, and health outcomes were documented in two different models of care involving persons in multiple professions when compared with independent solo physicians’ practices. In this commentary, we focus on the value of IP team-based care, continuing professional development, and the impact of the team on practice performance and health outcomes. Key components of effective IP teams include using consistent terminology to describe the team composition and function, team structures with purposeful selection of professions to address gaps in care, leadership support, and IP continuing professional development and education.

## Introduction

Receipt of preventive services is associated with a reduction in morbidity and mortality, most notably in the areas of cancer, chronic disease, infectious disease (immunizations), mental health, substance abuse, vision, and oral health [1]. A transition from disease treatment to prevention can reduce the incidence of chronic disease and decrease the cost of healthcare [2]. Despite the benefits of preventive care, limited numbers of individuals receive all recommended preventive services [2]. Literature on the rates of preventive service utilization is limited and varies based on population, age, and the type of preventive service. Disturbingly, a study by Borsky et al. reported that less than 8% of all United States adults received all recommended preventive care [3] .

Receipt of preventive services is influenced by a number of factors, including access to healthcare, cost, timeliness of recommendations by the primary care provider, and the capacity of the healthcare system to provide the needed care [1]. To help mitigate the barriers to obtaining preventive services, there is a growing interest in harnessing interprofessional (IP) teams in the delivery of clinical and preventive services [4, 5]. The IP team is well-positioned to address the complexities associated with prevention-related practice, population health management, care coordination, and access to healthcare.

A recently published study by Schor et al. [6] titled “Multidisciplinary work promotes preventive medicine and health education in primary care: a cross-sectional survey,” assesses and compares the provision of preventive and health education services in three primary care models in Maccabi Healthcare Services, Israel’s second largest healthcare organization. The purpose of their study was to examine the impact of a team approach on the provision and receipt of preventive services. Three models of care were compared. The Collaborative Model consisted of a physician and registered nurse; the Teamwork Model consisted of a dietician, physician, registered nurse, and social worker; and the Independent Practice Model consisted of a solo practicing physician. The Collaborative and Teamwork Models of care had higher rates of scheduling appointments proactively, participation by patients in health education groups, and better intermediate health outcomes such as occult blood testing, lipid levels, and influenza vaccinations when compared with independent physicians’ practices. Additional predictors of higher preventive medicine and health education implementation were health professionals’ occupation (nurses and dieticians) and health professionals’ personal health practices.

### Interprofessional team-based care and the delivery of prevention and health promotion services

Team-based care is not a new model for delivering primary care services. There is an increasing focus, however, on the design, implementation, and evaluation of IP healthcare teams and IP education with the goal of improving health outcomes and reducing costs. In recent years there has been a significant increase in publications and scholarship focusing on IP education and practice.

There is variation in the terminology used to describe team-based care and education. Terms being used include multidisciplinary, interdisciplinary, interprofessional, and multiprofessional. This variety of terms may lead to a lack of clear communication about the composition of the team and how the team interacts. This is demonstrated in the study by Schor et al. [6] in which the use of the term “multidisciplinary” was used; but the term was not defined and the interactions of the team members were not fully described or assessed. The Teamwork Model was composed of four professions but lacked team goals. Only physician goals were identified. Team members did not have a role in decision making related to clinic staff support; and only physicians received financial incentives for achieving defined outcomes. The Collaboration Model included a physician and nurse; however, the nurse was independent and consulted with the affiliated physician(s) on an as-needed basis. Although this sort of physician/nurse model is common in healthcare, it is not clear to what extent the physicians and nurses in the Collaboration Model perceived themselves as comprising a team with defined, shared goals.

In 2010, the World Health Organization and other leading international, interprofessional organizations adopted the term “interprofessional” [4, 7–11]. The World Health Organization defined interprofessional teams in the context of education, and their definition is also applicable to clinical practice. The term “interprofessional” applies when two or more professions learn or practice together to improve health outcomes [7]. By contrast, “multiprofessional” means more than one, but does not imply working together on shared goal(s) [4, 7, 12]. Interprofessional vs. multiprofessional or multidisciplinary practice more accurately describes what is desired in healthcare teams. Organizations such as the Interprofessional Education Collaborative (IPEC) provide standard IP operational definitions [4]. Use of consistent terminology and definitions helps support accuracy and consistency when developing, evaluating, and communicating about IP practice and education.

Two important criteria for sustainable and effective teams include good leadership and strong team structure. Within health systems, leadership has an important role in supporting IP teams [13]. Key characteristics include modeling and advocating for IP teamwork, providing resources and infrastructure (environment, staff, training, incentives, etc.), and promoting shared team leadership, goals and decision-making [13]. Schor et al. [6] noted the importance of leadership and organizational support in teamwork, engaging IP team members, providing trainings, and evaluating fee or incentive policies for performance.

In addition to leadership, the composition and structure of the team is critical for optimal performance and outcomes. In the Schor study [6], the professional disciplines represented on the Collaborative Model included a physician and nurse and the Team Model included physicians, nurses, dieticians and social workers. While the team structure, roles, and interactions were not evaluated in the study, the composition and roles of the interprofessional care team would ideally be determined by patient care needs. The first step when developing effective IP primary care teams is assessing the gaps in patient services and the needs of the patients [14]. The number and types of professions would optimally be deployed to address these gaps [14].

The IP team approach will benefit from professionals committed to supporting expanded roles, delegation of tasks, and overlap of roles. For example, advanced practice professionals [advanced practice registered nurses (APRN) and physician assistants (PA)] in the U.S. are able to perform health histories and physical exams; make diagnoses; and implement treatment plans designed to achieve high quality outcomes and patient satisfaction [15–17]. The integration of APRN and/or PA professionals on a team can increase access to care, reduce healthcare costs, and improve outcomes [15–17]. Registered nurses are also valued members of the primary care team and are enhancing services such as preventive care, chronic disease management, health education and transitions of care. Public health nurses, for example, play a central role in the provision of immunizations and other preventive services to infants and children receiving care in Israel’s Maternal and Child Health Clinics [18]. The opportunity for APRNs, PAs and registered nurses to practice to the full extent of their professional capability and capacity can enhance their job satisfaction and improve patient outcomes [19, 20]. Clinical pharmacists can also play a critical role in the provision of preventive services and the achievement of desired patient outcomes [21, 22]. In the U.S., clinical pharmacists can provide vaccinations, patient and provider consultation and education, medication management and reconciliation, quality improvement, and medication cost containment [21–23]. Other examples of professional disciplines that might be added to expand the range and quality of services made available to patients on primary care teams include behavioral health specialists, dentists, dental hygienists, and community health outreach workers.

An area that needs additional evaluation is the impact of financial payment models on the development and sustainability of IP teams and the provision of preventive services. Schor et al. [6] did not find an association between financial incentives and the implementation of preventive services. In her study, the Teamwork Model experienced higher implementation rates of preventive services without team monetary incentives when compared with the independent physician and Collaboration Model, both of which provided financial incentives. As Schor [6] acknowledges, the effect of payment models and financial incentives on the implementation of preventive services requires additional research. The current evidence is limited regarding the impact of incentives on quality metrics, preventive services, and IP team-based care [24–26]. Much of the research has focused on various physician incentives (i.e. pay for performance) with limited examination of alternative team-based payment models and the impact on IP teams, long-term sustainable behavior change, and adherence to guidelines and quality metrics. Future implementation and evaluation of incentive programs will need a focus on evaluating team-based incentive models, measurable outcomes, and sustainability to better understand the nuances and direct impacts of incentives on IP practice.

### Continuing professional development (CPD) and Interprofessional education (IPE)

Schor et al. [6] identified an association between preventive medicine training provided by the healthcare organization and the implementation of preventive measures and patient-directed health education tools. Professionals engaged in IP Teamwork and Collaboration Models who received preventive medicine training provided preventive medicine and health education services at a higher rate when compared with the independent physician model. Continuing professional development (CPD), i.e., education and training for clinicians in practice, helps to create and enhance skills, attitudes, and knowledge designed to maintain professional competency and quality of care, confidence in practice, and job satisfaction [27]. CPD specific to practice knowledge and skills, such as preventive services and population health, as well as IP teamwork competencies helps support collaborative practice-ready healthcare providers [4, 7]. In the United States, there is a heavy focus on engaging the pipeline of future healthcare providers in IP collaborative practice through IP education (IPE). IPE alone, however, does not translate into collaborative practice-ready professionals. IPE in the clinical setting is required as is continued CPD post-graduation. To build and support IP team skills, CPD on IP competencies is fundamental to enhancing IP team-based care in the primary care setting [4]. Specific to clinical knowledge and preventive services, the Clinical Prevention and Population Health Curriculum (CPPH) Framework is a resource that identifies key educational content to integrate into students’ curricula and CPD [5]. For example, Component 2 of the Framework, Clinical Preventive Services and Health Promotion, provides a structure for health professions education and CPD programs focusing on evidence-based health promotion and prevention services including lifestyle practices, approaches to behavioral change, identification of vulnerable populations, screening, behavioral health, immunizations, and preventive medicine [5]. Collaboration between the academic and practice settings in IP team-based competencies and clinical content can benefit patients, the team, and the practice. Building on the CPPH Framework, IP health professional students can add value to the primary care practice team as active and contributing team members who can help advance prevention-related practices and quality improvement initiatives designed to improve patient outcomes [4, 5].

### Assessing the impact of IP education and practice

In their study, Schor et al. [6] used the Maccabi Healthcare Service’s database to examine the extent of the provision of some preventive services (influenza immunization for high risk patients; lipids and fecal occult blood screening in low risk patients). The results of the preventive services provided by the Collaborative and Teamwork Models were statistically significant when compared with the solo physician practices, but the differences in the percentages between the three groups were not great. Schor et al. [6] also evaluated and found a positive correlation between health education CPD on the implementation of preventive services and the utilization of health education tools. Given the need for more evidence connecting IP education and teamwork with improved practice and patient outcomes, Schor and colleagues [6] are to be commended for the assessment of patient outcomes and health indicators [28, 29].

One important area for future study is the assessment of the effectiveness of IP CPD and preventive services on patients and healthcare systems using a mixed-methods approach. There is a need for a robust evaluation of not only the quantitative data on outcomes of preventive services provided, but also of qualitative information to guide the development of effective IP teams and to identify what teamwork strategies provide the greatest impact on outcomes [28]. Qualitative evaluation helps to expand knowledge in the areas of implementation experiences, institutional commitment, and sustainability [30]. Areas worthy of future study include quantitative outcomes that focus on health (not just on healthcare quality indicators), patient satisfaction, healthcare provider satisfaction, and the impact of IP practice on return on investment. An IP conceptual model for evaluating the impact of health outcomes will be important to guide future study design and strengthen the evidence for IP practice [28]. The Interprofessional Learning Continuum (IPLC) model is an IP conceptual model developed by the Institute of Medicine to support the design and evaluation of IP education and practice initiatives. The model outlines the IP learning continuum from academic education through to clinical practice and the impact on learning, patient, and health system outcomes [28].

Other areas for future research include: the development and comparative function of IP teams composed of different professional disciplines such as APRNs, PAs and pharmacists [31]; the impact of key IP principles such as team responsibilities for developing and meeting established goals, contributing to decisions about staff and team composition, and rewards for achieving the established goals; development and evaluation of IP teams that emphasize patient engagement when establishing care goals and making team decisions.

## Conclusion

Healthcare delivery in coming years will increasingly rely on primary care and an emphasis on prevention. Access to quality primary care and preventive health services is associated with improved health outcomes and lower costs. As revealed in the study by Schor et al. [6], more preventive services were delivered by IP primary care teams when compared with independent physician practices. The provision of primary and preventive care is a complex and multifaceted process with a focus on improving health outcomes and reducing health disparities for individuals, populations, and communities. Successful IP primary care teams with disciplines that meet the needs of those they serve will benefit from having a collective identity and shared responsibilities and goals. As care models change and flex to meet the needs of individuals and populations, the healthcare workforce must be able to adapt. Retooling the current healthcare workforce while educating the future workforce requires flexibility in provider roles and continued IP education [14]. Providing healthcare professionals and students with knowledge of IP competencies and continuing education on prevention and population health will help prepare the primary care workforce for the future [4, 5]. Although further evaluation of the impact of IP team-based care on the provision of primary care and preventive services will be important, Schor and her colleagues [6] have made a valuable contribution by examining some of these issues in their publication.

## Data Availability

Not applicable.

## Abbreviations

APRN: Advanced practice registered nurses
CPD: Continuing professional development
IP: Interprofessional
IPE: Interprofessional education
IPEC: Interprofessional Education Collaborative
IPLC: Interprofessional Learning Continuum
PA: Physician assistants
